# Overstimulation can create health problems due to increases in PI3K/Akt/GSK3 insensitivity and GSK3 activity

**DOI:** 10.1186/2193-1801-3-356

**Published:** 2014-07-14

**Authors:** Xunxian Liu

**Affiliations:** Intramural Research Program, National Center for Complementary and Alternative Medicine, US Department of Health and Human Services, National Institutes of Health, Bethesda, MD 20892 USA

**Keywords:** Aging, AMD, Complement, Death hormones, Insensitivity, IL17/IL17RC, PI3K/Akt/GSK3/GSK3 substrates, Signaling, VEGF

## Abstract

Aging is linked to decrease of the body cell use of growth hormone (GH) and thyroxine, whereas the decrease is via “death hormones” inhibition? This study proposes different viewpoints. Since interleukin 17 receptor C (IL17RC) is highly expressed in tissues from age-related macular degeneration (AMD) patients, IL17RC signaling pathways are explored to evaluate Wnts/vascular endothelial growth factor (VEGF) expression and complement activity, which are pathological factors in AMD. IL17RC overexpression or VEGF treatment was performed in two cell lines for up to two-day. Real-time Quantitative PCR, confocal microscopy, immune-blot, MTT assay, etc. measured downstream effects. IL17RC overexpression increases Wnts and VEGF that forms complexes with Wnt-signaling components. VEGF or the Wnt-signaling components interacting with C3 suggests alternative complement pathway activation. Moreover, IL17RC-overexpressed cells or VEGF-treated cells for two-day, which is overstimulation, increase PI3K/Akt/GSK3 insensitivity and GSK3 activity, and decrease growth/survival. High GSK3 activity associates with many chronic diseases including type II Diabetes. This study shows high GSK3 activity can result from PI3K/Akt overstimulation. Type II Diabetes shows insulin resistance that the body cells decrease insulin use. Possessing little sensitive PI3K/Akt for receptor activation, cells after overstimulation, although live, hardly respond to PI3K/Akt activators including GH, thyroxine and insulin. These results suggest an alternative explanation of the body cells declining hormone use since various kinds of cell signaling-induced overstimulation events almost always linked to PI3K/Akt, increase with age. Playing pathological roles in senescence and diseases, overstimulation eventually generates health problems.

**Electronic supplementary material:**

The online version of this article (doi:10.1186/2193-1801-3-356) contains supplementary material, which is available to authorized users.

## Introduction

In 1974, Dr. W. Donner Denckla proposed Death Hormone or DECO (decreasing oxygen consumption hormone) Theory (Denckla 1974). Regularly released by the human pituitary gland, DECO suppresses the ability of cells to use thyroxine (Denckla 1974), which causes people to age. The principal functions of thyroxine are to stimulate oxygen consumption and control metabolism of all cells and tissues in the body (Welcker et al. 2013). Age-related decrease in use of growth hormone (GH) by body cells has also been found (Blackman et al. 2002). However, the death hormones are nonetheless unidentified. Because of high expression of IL17RC in peripheral blood and chorioretinal tissues with AMD lesions increases age-related macular degeneration (AMD) risk (Wei et al. 2012), exploration of IL17RC signaling pathways sheds light on possible molecular biology mechanisms that reduce the use of the relevant hormones in the body cells. These mechanisms lead to an alternative proposal of the Death Hormone Theory.

AMD is the leading cause of irreversible blindness in the elderly population worldwide. The disease causes progressive loss of central vision because of macular geographic atrophy (GA) (dry AMD) or choroidal neovascularization (CNV) (wet AMD) (Sunness 2006). AMD is associated with many factors. A recent study shows that higher levels of interleukin 17 receptor C (IL17RC) are detected on the surface of peripheral blood cells from AMD patients (Wei et al. 2012). How high expression of IL17RC increases AMD risk, which is investigated in the study, is unknown. High expression of IL17RC is also detected in the chorioretinal tissues containing AMD lesions (Wei et al. 2012), whereas GA or CNV of the retinal pigment epithelium (RPE) results in RPE cell death in the two forms of advanced AMD (Sunness 2006). Therefore, in the study, the experiments were performed using cultured cells: ATCC retinal pigment epithelium (ARPE) and the culture of monocytes (THP-1) without or with high expression of IL17RC. This research suggests that the etiology of AMD associated with high expression of IL17RC (Wei et al. 2012) is attributed to two pathways: increased complement and GSK3 activities that cause cell death. Although the original objective was to find how high expression of IL17RC increases AMD risk, this study had three findings in general cell signaling: 1. overstimulation causes insensitivity of PI3K/Akt/GSK3 that is associated with almost any cell signaling; 2. releases GSK3 activity; and 3. can be created by PI3K/Akt activators. These findings are the basis for an alternative explanation of declined use of the hormones in the body cells.

## Materials and methods

### Cells and transfection

ARPE (RPE from ATCC) and THP-1 cultured monocytes) were obtained from ATCC, and the cells were cultured according to the protocols from ATCC. Transfection (2 μg of empty vector (EV) or 2 μg of IL17RC) was performed using Nucleofector II purchased from Lonza according to the protocols from the manufacturer.

### Antibodies and reagents

Anti (α)-Akt, α-pAkt, α-Axin, α-β-catenin, α-C3, α-c-Myc, α-cyclin D1, α-GSK3, α-GSK3β, α-pGSK3, α-IL17, α-IL17RC, α-IRS1, α-PI3K, α-pPI3K, α-TAU, α-VEGF, α-Wnt-3a, and α-Wnt-10b were purchased from Santa Cruz. α-c/EBPα, α-pc/EBPα, α-ERK1/2, α-pERK1/2, and α-pIRS1 were bought from Cell Signaling Technology. α-GSK3β was obtained from BD Transduction Lab. GAPDH was purchased from ImmunoChemical. IL17A, IL17F and sFRP2 were bought from R and D. VEGF was obtained from Sigma-Aldrich. IL17A or IL17F (500 ng/mL), sFRP2 (250 ng/mL) or VEGF (10 ng/mL) was applied to cell culture. An IL17RC expression vector was purchased from OriGene.

### Confocal microscopy analysis

**Figure 1:**
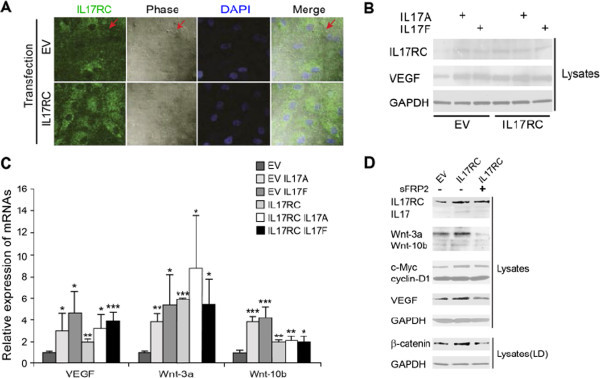
**IL17RC induces VEGF and Wnts. A**. Confocal microscopy procedures were applied to EV- or IL17RC-transfected ARPE. Images are indicated in the figure and an example spot is indicated by arrows. DAPI is the intensity control and phase shows cell-surface. **B**. EV- or IL17RC- transfected ARPE cells were treated as indicated. Cell lysates were immune-blotted with indicated antibodies. **C**. RT-PCR of VEGF, Wnt-3a and Wnt-10b. EV- or IL17RC- transfected ARPE cells were treated as indicated. Values of mRNA from EV are set as one. Data are averaged from three or more separate experiments and represented as mean ± SD. *, ** or *** versus EV of the cognate groups: p < 0.05, 0.01 or 0.001. **D**. EV- or IL17RC- transfected ARPE cells were treated as indicated. Cell lysates were immune-blotted with indicated antibodies. LD: low concentration of detergent. In **B** and **D**, GAPDH is the loading control. For **A**, **B** and **D**, data represent three or more separate experiments. Statistic details of the blots in **B** or **D** are shown in Additional file 1: Figure S1A or B.

**Figure 2:**
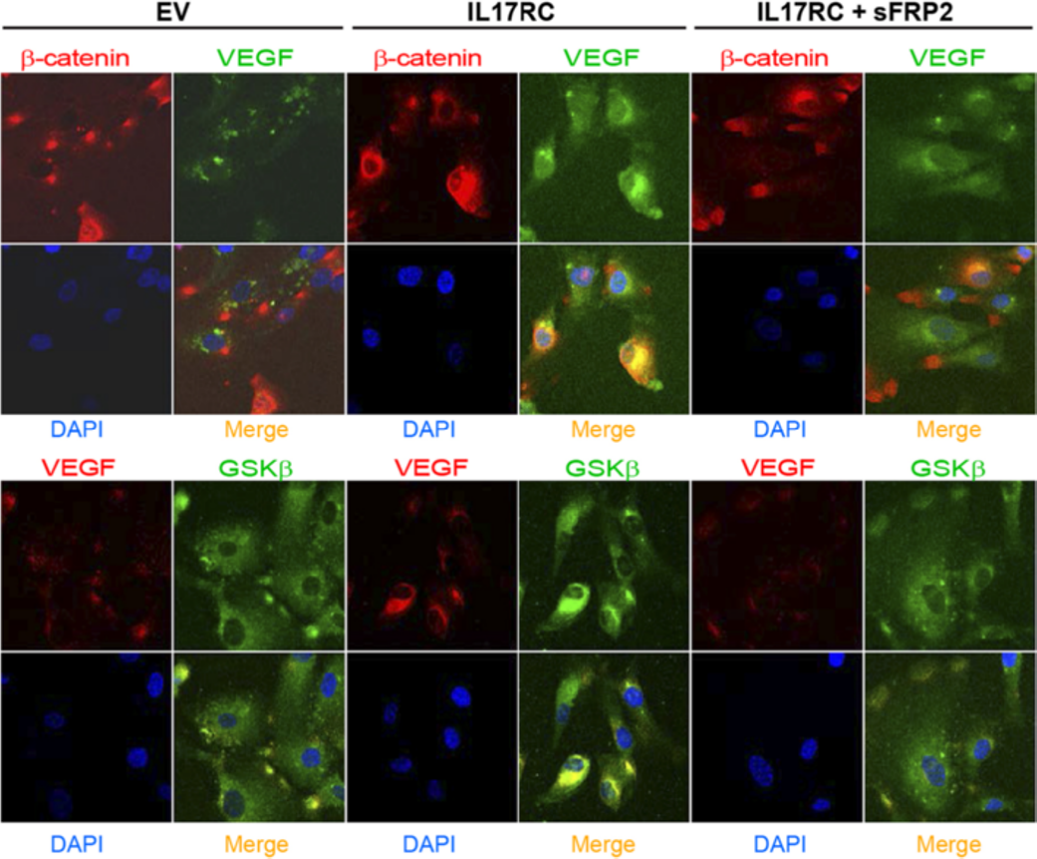
**IL17RC overexpression increases colocalization of VEGF/β-catenin and VEGF/GSK3β.** Confocal microscopy procedures were applied to EV-transfected ARPE or IL17RC-transfected ARPE treated without or with sFRP2. Images were indicated in the figures. DAPI is the intensity control. Data represent three separate experiments.

**Figure 3:**
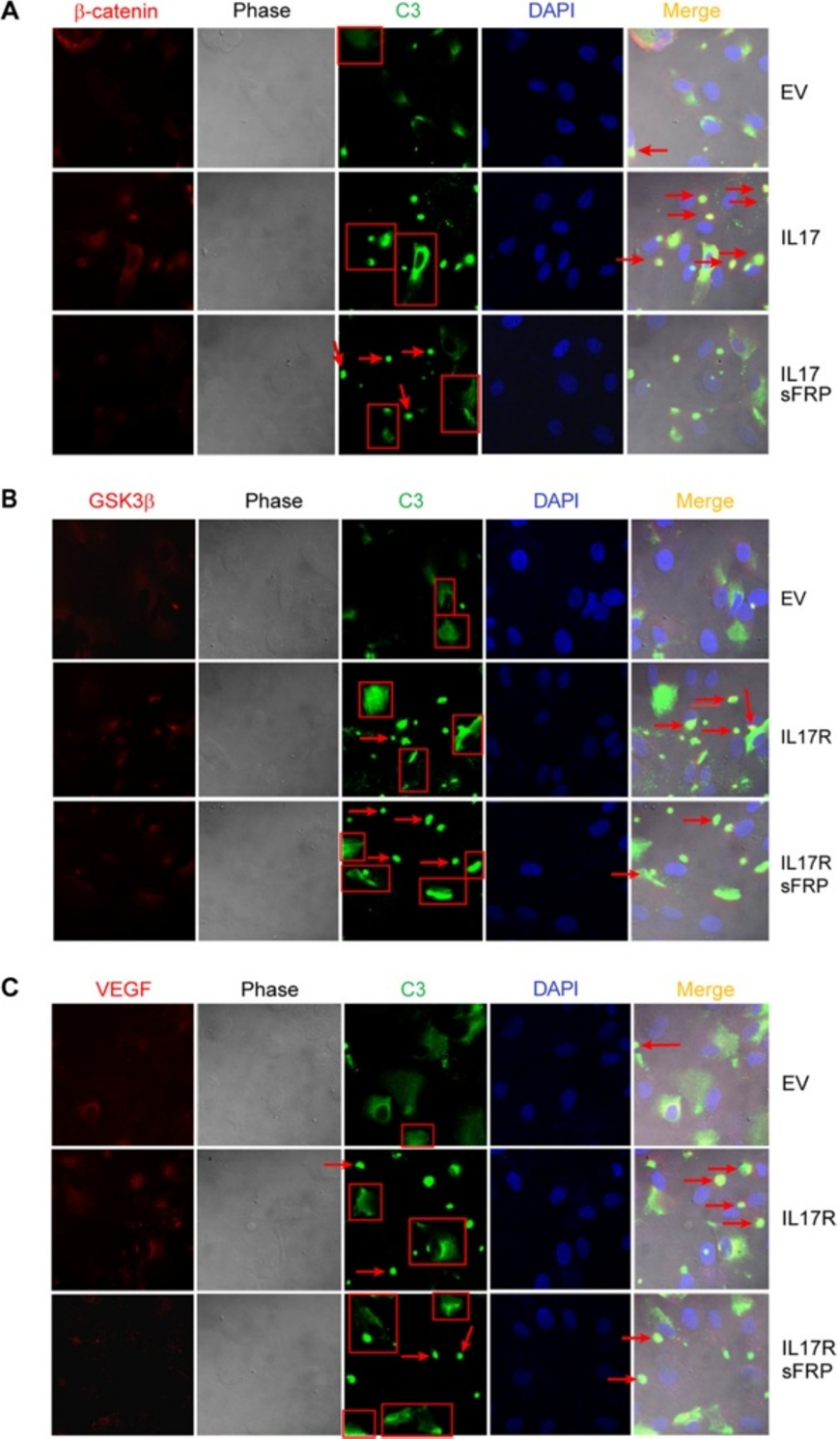
**IL17RC overexpression increases complexes of C3/β-Catenin, C3/GSK3β or C3/VEGF.** In **A** to **C**, confocal microscopy procedures were applied to EV-transfected ARPE or IL17RC-transfected ARPE treated without or with sFRP2. Images and treatments are labeled in the figures, big C3 complexes are indicated by arrows, and damaged cells are red-framed. DAPI is the intensity control and phase shows cell-surface. Data represent three separate experiments.

EV- or IL17RC-transfected ARPE cells were plated onto sterile cover slips. Cells were left untreated or treated with sFRP2 for two-day. The fixing, blocking and staining procedures have been described (Liu et al. 2011), but the buffers did not contain any detergent. Samples were stained with primary rabbit antibodies 1:50 (α-IL17RC for Figure 1A, α-β-catenin for Figures 2 and 3A, α-VEGF for Figure 2, the bottom panel and Figure 3C, α-GSK3β for Figure 3B) and primary mouse antibodies (α-VEGF for Figure 2, the top panel, α-GSK3β for Figure 2, α-C3 for Figures 3A-C), as well as secondary antibodies goat anti-rabbit conjugated FITC 1:100 for Figure 1A or donkey/goat anti-rabbit conjugated rhodamine 1:100 combining with goat anti-mouse IgG1 conjugated FITC at 1:100 for Figures 2 and 3. The secondary antibodies were purchased from Santa Cruz. VECTA-SHIELD HardSet Mounted Medium with DAPI (Vector Labs) was employed for mounting cover slips. Cells were viewed with a confocal laser microscope (Lenses: 63 W;LSM510; Carl Zeiss MicroImaging, Inc.) in the Lab of Cellular and Developmental Biology, NIDDK, NIH.

### Western blot analysis

Cultured cells were lysed with lysis buffer from Cell Signaling Technology, and lysates were cleared at 3000 rpm and 4°C or treated ARPE was lysed with a buffer containing a low concentration of detergent for detection of β-catenin (Figure 1D). The buffer and the procedure for preparation of lysates with a low concentration of detergent have been described in (Liu et al. 2011). Protein concentrations were assessed in the cell lysates using bicinchoninic acid protein (BCA) assay kit (Thermo Scientific). Western blotting procedures have been described (Liu et al. 2011; Liu et al. 2005). Densitometer to scan Western blot bands was NIH Image J.

### Real-time quantitative PCR (RT-PCR) analysis

EV- or IL17RC-transfected ARPE cells were plated 2000 cells/well in a 96-well plate or 20000 cells/well in a 24-well plate. Cells were left untreated or treated with IL17A or IL17F for two days. Power SYBR Green Cell to CT Kit (Applied Biosystems) was directly applied to cells in the 96-well plates according to the protocol from the manufacturer to generate RT-PCR results, or RNA was extracted from cells in 24-well plates using a Qiagen kit. Primers to amplify VEGF, Wnt-3a, Wnt-10 and RPLP0 are described as follows: VEGF forward 5′ctacctccaccatgccaagt, reverse 5′tggtgatgttggactcctca; Wnt-3a forward 5′caagattggcatccaggagt, reverse 5′atgagcgtgtcactgcaaag; Wnt-10b forward 5′tgctttttccttctccatgc, reverse 5′tccaagaaatcccgagagaa; RPLP0 forward 5′ ggcgacctggaagtccaact, reverse 5′ ccatcagcaccacagccttc. RT-PCR procedures from total RNA have been described (Liu et al. 2011). Each value of mRNAs was averaged from means, determined by three to six replicates and normalized by RPLP0 mRNA values in three or more separate experiments. Gene expression in untreated EV-transfected cells was used as a control to compare with that in cells with any other treatment.

### MTT assay analysis

**Figure 4:**
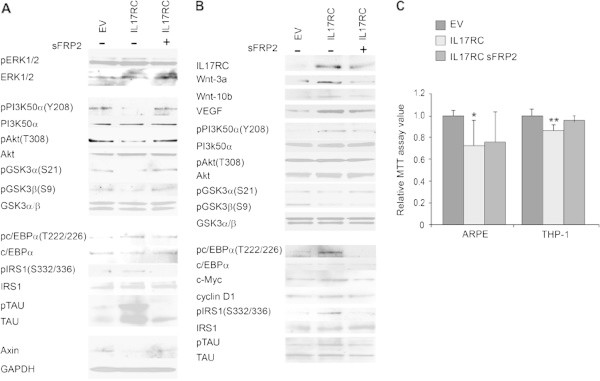
**IL17RC induces insensitivity of PI3K, Akt or GSK3.** EV-transfected ARPE **(A)** or THP-1 **(B)** or IL17RC-transfected ARPE **(A)** or THP-1 **(B)** treated without or with sFRP2, cell lysates were immune-blotted with indicated antibodies. In **A**, GAPDH is the loading control; data represent three or more separate experiments. **C**. MTT assay of EV-transfected cells or IL17RC- transfected ARPE or THP-1 cells treated without or with sFRP2. Data are averaged from three or more separate experiments and represented as mean ± SD. * or ** versus EV in the cognate groups: p < 0.05 or 0.01. Statistic details of the blots in **A** or **B** are shown in Additional file 1: Figure S1C or Additional file 2: Figure S2A.

**Figure 5:**
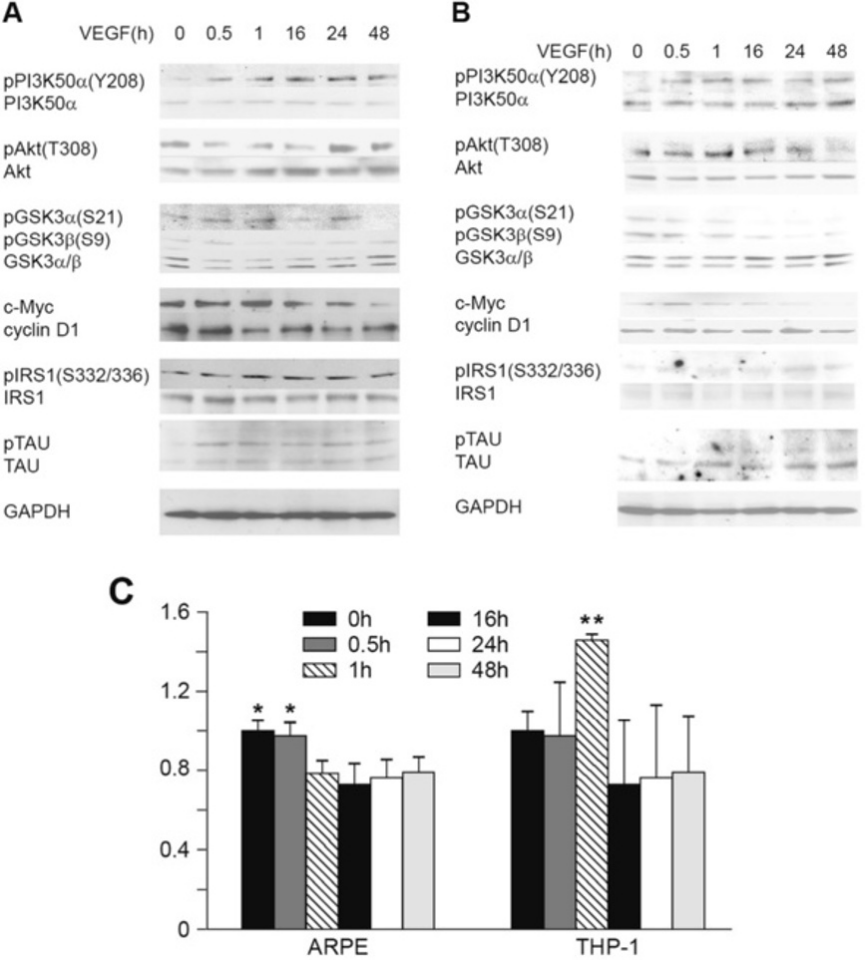
**VEGF induces insensitivity of Akt or GSK3.** VEGF-treated ARPE **(A)** or THP-1 **(B)** cell lysates were immune-blotted with indicated antibodies; GAPDH is the loading control; data represent three or more separate experiments. **C**. Results of MTT assay (growth/survival). Data are averaged from three or more separate MTT assay experiments, and represented as mean ± SD. * or ** versus any other treatment: p < 0.05 or 0.01. Statistic details of the blots in **A** or **B** are shown in Additional file 2: Figure S2B and C or Additional file 3: Figure S3A and B.

MTT assay kit was purchased from Promega, and the assay was performed according to the manufacturer protocol. For Figure 4C, MTT assay of 8 or more replicates (EV- or IL17RC-transfected cells) was performed. The values were measured at day 0. Two days later, MTT assay of 10 or more replicates (EV-transfected cells or IL17RC-transfected cells treated without or with sFRP2) was performed. The values of day 2 divide those of day 0 in the cognate groups. The ratios from EV groups are set as one. For Figure 5C, values of each time point were determined by 8 or more replicates and the value at 0 h was set as one.

### Statistical analysis

All shown data represent three or more experiments. Quantitative data are presented as mean values ± SD, averaged from three or more separate experiments. The statistical analysis was performed using the JMP (SAS Institute) statistical package based on t-test. An adjusted P-value of < 0.05 was considered significant.

## Results

### IL17RC overexpression increases expression of canonical Wnts and Wnt-downstream target gene and proteins in ARPE

Because high expression of IL17RC protein in peripheral blood and chorioretinal tissues with AMD lesions (Wei et al. 2012), transfection of IL17RC in cultured cells was performed to achieve high IL17RC expression. EV or IL17RC was transfected into ARPE for two-day. IL17RC was partly expressed on the cell surface as an example spot that was stained green in the green image and that topped the cell surface in the phase image as indicated by the arrows. The green spot superimposes the phase spot in the merge image (Figure 1A). IL17RC-overexpressed ARPE had higher expression of IL17RC using confocal microscopy because these cells were stained greener (Figure 1A). Since Wnt-signaling plays pathogenic roles in AMD in a mouse model and Wnt-3a induces vascular endothelial growth factor (VEGF) in ARPE (Zhou et al. 2010), protein levels of IL17RC and VEGF as well as mRNA levels of VEGF and Wnts in ARPE were examined. More protein expression (2 or more folds) of IL17RC and VEGF as well as more mRNA expression (2 or more folds) of VEGF, Wnt-3a and Wnt-10b were detected in IL17A or IL17F-treated and/or IL17RC-transfected ARPE than those in EV-transfected ARPE. However, IL17A or IL17F did not further increase the protein and/or gene expression levels in IL17-RC transfected ARPE (Figures 1B and C), probably due to the system saturation in the same pathway or insensitivity to long strong stimuli (see below) such as overexpression of IL17RC and/or treatment of IL17A or IL17F in ARPE for two-day. Because Wnts were induced by IL17RC overexpression, Wnt-signaling was tested. More protein expression (> 2-fold increase) of Wnt-3a and Wnt-10b, Wnt-target genes or stabilized protein (VEGF (Zhang et al. 2001) (> 2-fold increase), c-Myc (He et al. 1998) (> 2-fold increase), cyclin D1 (Tetsu and McCormick 1999) (> 20% increase), and β-catenin (Liu et al. 2005) (> 2-fold increase) was detected in IL17RC-overexpressed ARPE than that in EV-transfected ARPE, whereas secreted frizzled-related protein 2 (sFRP2) abolished most of the induction levels in comparison to the levels of those proteins in IL17RC-transfected ARPE (Figure 1D). sFRPs act as soluble inhibitors of Wnt signaling (Rattner et al. 1997) and sFRP2 did not decrease IL17RC overexpression (Figure 1D). Despite expectation of higher levels of Wnt-3a and Wnt-10b, the lowest levels of those were detected in IL17RC-overexpressed and sFRP2-treated ARPE (Figures 1D) perhaps because, when associated with sFRP2 in media, Wnts could no longer be bound to their receptors on the cell surface or be detected by immune-blots. Interestingly, protein levels of IL17RC were increased by treatment of IL17A or IL17F (Figure 1B), whereas endogenous IL17 was also increased by overexpression of IL17RC (Figure 1D). These data suggest the cognate ligand/receptor interactions, resulting in decrease of the protein degradation. This hypothesis is partly supported by IL17-increased gene expression of IL-17RA but not IL17RC (Venkatachalam et al. 2008).

### IL17RC overexpression increases interactions of VEGF/β-catenin and VEGF/GSK3β in ARPE

Although VEGF gene and protein expression was increased in IL17RC-transfected ARPE (Figures 1B and C), VEGF interaction with Wnt-signaling components for contribution to increased VEGF protein expression in IL17RC-transfected ARPE (Figure 1D) was sought. Confocal microscopy detected more pure yellow fluorescence in the merge images from IL17RC-transfected ARPE than in those images from EV-transfected ARPE, showing that co-localization of VEGF/β-catenin and VEGF/GSK3β is increased but that the increased levels were abrogated by sFRP2 treatment (Figure 2). Therefore, Figure 2 suggests that higher interactions of VEGF/β-catenin and VEGF/GSK3β contribute to higher protein expression of VEGF in IL17RC-overexpressed ARPE.

### IL17RC overexpression increases alternative complement pathway in ARPE

Since VEGF or β-catenin can be located outside cells or in plasma membrane, whether β-catenin, GSK3β or VEGF is associated with complement was investigated in that the interactions of VEGF/β-catenin and VEGF/GSK3β (Figure 2) are aberrant and possible on the cell surface. These interactions were also accompanied with higher levels of the protein expression, such as β-catenin and VEGF (Figure 1), whereas higher protein expression augments possibility to produce abnormal proteins. Complement also plays the pathogenic role in AMD (Zipfel et al. 2010). Alternative complement pathway was investigated since it is activated by foreign antigens/abnormal proteins on the cell surface (Abbas et al. 2010), whereas activation of classical complement pathway requires antibodies (Abbas et al. 2010) and it is impossible to generate antibodies *in vitro* and in two-day. The first activated component of alternative complement pathway by foreign antigens/abnormal proteins is C3 (Abbas et al. 2010). Confocal microscopy detected C3 (green) co-localized with β-catenin, GSK3β or VEGF (all are red) in distinct compartments (Figure 3). Big green or yellow spots are indicated by red arrows in green or merge images (Figures 3A-C). Those spots are considered to be C3 complexes because: 1) they are outside cells; 2) some of the spots are located on damaged cells with distorted nuclei (DAPI, blue) or cell shapes, suggesting that the complexes are cell killers. However, most of the spots were located on intact cells since C3-activated complements-pathway is inhibited by complement factor H/I (Abbas et al. 2010) or the complement complex to destroy cells might be in early stages. Small green spots are not counted as they might be inactivated C3 complexes or their complement reactions might be incomplete. Moreover, damaged cells are included by red frames. Often accompanied by bright C3 staining, those damaged cells include cells with distorted or shrunken nuclei, distorted cell shapes, or cells without nuclei. By either of the criteria, IL17RC overexpression increased alternative complement pathway activity, compared with that in EV-transfected ARPE (Figure 3). Despite blocking IL17RC-induced Wnt-signaling and interactions of VEGF/β-catenin and VEGF/GSK3β (Figures 1 and 2), sFRP2 treatment did not reduce the complement activity, compared with that in IL17RC-transfected ARPE (Figure 3), perhaps because recombinant mouse sFRP2 is a foreign protein for human ARPE. Nevertheless, the images show most arrow-indicated spots in the merge images from IL17RC-transfected cells because β-catenin, GSK3β or VEGF was in the C3 complexes indicated by more or less yellow fluorescence; however, comparable amounts of the arrow-indicated spots are observed in the green images from IL17RC-transfected and sFRP2-treated ARPE, which shows that the C3 complexes (green spots) did not contain β-catenin, GSK3β or VEGF. The framed damaged cells in the images from IL17RC-transfected ARPE also show the most co-localization of C3 with β-catenin, GSK3β or VEGF (Figure 3). Figures 2 and 3 suggest that IL17RC-induced Wnt-signaling and VEGF are involved in the activation of complement.

### IL17RC overexpression causes PI3K/Akt/GSK3 insensitivity and cells death

As VEGF was increased in IL17RC-transfected ARPE (Figure 1), and VEGF belongs to growth hormone family in which a member generally activates extracellular signal-regulated kinases (ERK1/2) and PI3K/Akt (Liu et al. 2011), the signaling pathways were tested. Figure 4A and B shows that phosphorylation of ERK1/2, especially ERK1, was increased. However, tyrosine phosphorylation (Y208) of PI3K50α (pYPI3K) (Accession#:NP_852665.1), one of PI3K regulatory subunits, was surprisingly abrogated in cells transfected with IL17RC for two-day. Contrasting with the above statement (VEGF activating PI3K/Akt), the observation suggests that PI3K is insensitive to persistently high levels of VEGF and/or other PI3K stimuli. Since PI3K activates Akt and activated Akt inhibits GSK3 (PI3K Kinase Akt Signaling, Cell Signaling Technology), inactivation of PI3K decreased Akt activity because of decreased detection of threonine phosphorylation (T308) (pSAkt), leading to increase of GSK3 activity due to lower levels of serine phosphorylation (S21 and S9) (pSGSK3) in IL17RC-transfected cells (Figure 4A). Consequently, phosphorylation levels of GSK3 substrates, CCAAT-enhancer-binding protein α (Ross et al. 1999), (pcEBPα), Insulin receptor substrate 1 (pIRS1) (Liberman and Eldar-Finkelman 2005), and Tau protein (pTAU) (Hanger et al. 1992) increased, compared with those in cells transfected with empty vector (EV); sFRP2 treatment abolished the effects induced by IL17RC transfection (Figure 4A). Immune-blots of all the total proteins, in addition to measurement of levels of the phosphorylation proteins, were also performed (Figure 4A). These data show higher GSK3 activity in ARPE transfected with IL17RC for two-day. However, β-catenin is also a GSK3 substrate (Ikeda et al. 1998) but may not be a Wnt-target gene as there is no such report, and it is still stabilized (Figure 1D) in the environment with high GSK3 activity, probably due to Wnt-induced Axin degradation (Figure 4A). GSK3 phosphorylating β-catenin requires Axin (Liu et al. 2002; Amit et al. 2002).

Similar results were observed in IL17RC-transfected THP-1. After cells were transfected with IL17RC for two-day, Wnt-3a, Wnt-10b and VEGF were increased (Figure 4B). In contrast with the insensitivity target, PI3K in ARPE (Figure 4A), the insensitivity targets to the regulation were identified to be Akt and GSK3 in IL17RC-transfected THP-1 because the level of pYPI3K was increased, whereas the level of pSAkt was unchanged, and the levels of pSGSK3 were lowest in comparison to those in EV-transfected cells and IL17RC-transfected plus sFRP2-treated cells (Figure 4B). Nevertheless, the patterns of phosphorylation levels of GSK3 substrates in THP-1 cells were similar to those in ARPE cells (Figures 4A and B).

GSK3 participates in various apoptotic signaling pathways by phosphorylating transcription factors regulating apoptosis (Jope and Johnson 2004); GSK3 promotes apoptosis by both activating pro-apoptosis factors such as p53 (Watcharasit et al. 2002) and inactivating survival-promoting factors through phosphorylation (Grimes and Jope 2001). Cell growth/survival by transfection of IL17RC was tested. MTT assays show that overexpression of IL17RC decreased cell growth/ survival in ARPE (p < 0.05) and THP-1(p < 0.01) (Figure 4C), which is consistent with that high GSK3 activity induces cell apoptosis (Jope and Johnson 2004; Watcharasit et al. 2002; Grimes and Jope 2001). Despite insignificant rescues, sFRP2 treatment decreased a little death in IL17RC-overexpressed ARPE and more death in IL17RC-overexpressed THP-1 because the sFRP2 is a foreign protein for human cells and can activate complement alternative pathway (Figure 3). Additionally, the GSK3 insensitivity percentages, (100% pSGSK3αβ levels in EV-transfected cells) - % of pSGSK3αβ levels in IL17RC-transfected cells) (Additional file 1: Figure S1C and Additional file 2: Figure S2A), were about 56% in ARPE and 44% in THP-1, compared with those insensitivity percentages (set as 0%) of the cognate cells transfected with EV. Therefore, IL17RC overexpression causes more than 50% of damage in these cells by decreasing growth/ survival and increasing GSK3 insensitivity by adding the percentages of growth/survival decrease (Figure 4C) and GSK3 insensitivity that was rescued by sFRP2 treatment (Additional file 1: Figure S1C, Additional file 2: Figure S2A).

### VEGF causes Akt/GSK3 insensitivity and cells death

To further investigate the concept that PI3K/Akt/GSK3 insensitivity to regulation can be induced in cells by long treatment of VEGF alone, cells were merely administrated with VEGF to eliminate the complex of stimuli as IL17RC signaling and/or Wnt-signaling induce expression of a number of genes that may result in other PI3K stimulators in addition to VEGF. Kinetics of pYPI3K, pSAkt, pSGSK3 and phosphorylation of GSK3 substrates as well as growth/survival in ARPE or THP-1 was tested. Cells were treated with VEGF at 0, 0.5, 1, 16, 24 and 48 h. Treated cell lysates were collected for Western blots, or the treated cells were applied to MTT assays. Figure 5A shows that the insensitivity target proteins to the regulation were identified to be GSK3 because the levels of pYPI3K and pSAkt were elevated, whereas the levels of pGSK3 were significantly reduced at the later time points. Moreover, phosphorylation levels of GSK3-target substrates were suggested to have increased as the protein expression levels of c-Myc and cyclin D1 were decreased, whereas the levels of pIRS1 and pTAU were increased at later time points in ARPE (Figure 5A). Figure 5B shows that the upstream insensitivity target protein to the regulation was Akt as the levels of pYPI3K consistently increased in comparison to those at time 0. However, the levels of pSAkt significantly increased at 1 h (p < 0.05) and 16 h (p < 0.01) (Additional file 3: Figure S3C) compared to those at 0 h, whereas those levels were decreased at later time points (Figure 5B). GSK3 insensitivity to Akt regulation was also observed because of the highest level of pSGSK3 at time 0 (Figure 5B and Additional file 3: Figure S3C). Similar phosphorylation patterns of GSK3 substrates in THP-1 to those in ARPE were detected (Figures 5A and B). In both systems, there was no additional Wnt-signaling so that the degradation of c-Myc and cyclin D1 was apparently detectable further verifying that GSK3 activity was increased by read-out from the GSK3-target substrates at the later time points (Figures 5A and B). MTT assays show that growth/survival in ARPE was decreased at the later time points (1 h to 48 h) (p < 0.05). The maximum values of growth/survival and pGSK3 which was the insensitivity target in VEGF-treated ARPE were detected at around 0.5 h (Figures 5A and C). Although MTT assays show that growth/survival in THP-1 did not significantly decrease at 48 h compared with that at 0 h, the growth/survival significantly increased at 1 h compared to that of any other time point (Figure 5C) (p < 0.01). The maximum values of growth/survival and pAkt which was the insensitivity target in VEGF-treated THP-1, were detected at approximately 1 h (Figures 5B and C). Hence, the results suggest that growth/survival is determined by the upstream insensitivity target in cells treated with VEGF for a while. The GSK3 insensitivity percentages (100% (pSGSK3αβ levels at 0 h) - % of pSGSK3αβ levels at 48 h) (Figure 5A and B) are over 50% in ARPE or over 70% in THP-1. Including the percentages of growth/survival reduction and GSK3 insensitivity, more than 70% of damage in ARPE or THP-1 was caused by VEGF treatment for 48 h (Figure 5).

## Discussion

**Figure 6:**
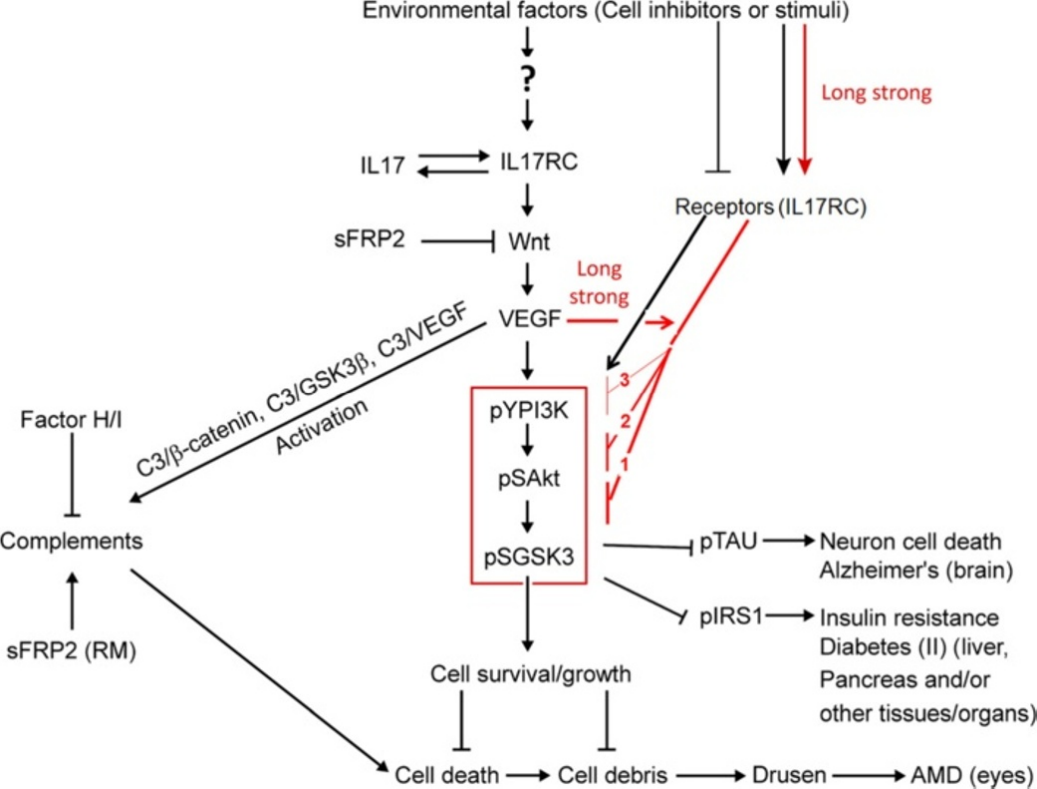
**Potential pathways tested or stated in the study lead to diseases including AMD.** Details of the pathways are depicted in the text. sFRP2 (RM): recombinant mouse sFRP2. Red arrows and blocks: provided that the stimuli are overstimulation of PI3K/Akt, which is the improvement of the doctrine. The numbers 1, 2, 3 in the block lines represent insensitivity stages 1(GSK3 insensitivity), 2 (Akt insensitivity), 3 (PI3K insensitivity). PI3K/pSAkt/pSGSK3 is put into the red frame. The black arrows between VEGF and PI3K, and stimuli and receptors indicate stimulation without causing insensitivity or high GSK3 activity.

In this study, the evidence suggests that IL17RC increases AMD risk via two pathways, alternative complement pathway and insensitivity of PI3K/Akt/GSK3 (delinked regulation by stimuli) resulting in increased GSK3 activity (Figure 6). Both pathways can be induced by IL17RC-increased Wnt-signaling and VEGF, whereas sFRP2 blocks IL17RC-induced insensitivity due to its inhibitory effects on Wnt-signaling (Figures 1, 4 and 6). Since high levels of IL17RC are detected in chorioretinal tissues with AMD lesions (Wei et al. 2012), this study shows that high IL17RC expression increases complement and GSK3 activities, resulting in less cell growth/survival (Figures 3 and 4). Moreover, Th17 cells are essential in clearing pathogens during host defense and in inducing inflammation during autoimmune disease (Korn et al. 2009). Elevated levels of IL17A are found in AMD patients’ serum and macular tissues and high IL17RC levels are detected in CD14^+^ monocytes from AMD patients, whereas high levels of IL17 increases IL17RC and *vice versa* (Figure 1), and high IL17RC levels increase GSK3 activity in cells (Figure 4); therefore, living in the same environment as CD14^+^ monocytes, Th17 cells in AMD patients are suggested to have higher levels of GSK3 activity, resulting in apoptosis (Jope and Johnson 2004; Watcharasit et al. 2002; Grimes and Jope 2001), and to produce more inactive proteins by GSK3 phosphorylation. These proteins, such as transcription activators (Jope and Johnson 2004), survival-promoting factors (Jope and Johnson 2004; Grimes and Jope 2001) and IRS1 (Liberman and Eldar-Finkelman 2005), are required for normal cell functions. Hence, high IL17RC expression in eye and peripheral blood cells (Wei et al. 2012) increases rate of cell death and decreases rate of debris clean in eye cells, leading first to accumulation of drusen in macula and then to AMD. This research reveals relationships (Figure 6) among IL17RC, Wnt-signaling/VEGF and complement, which have been thought or treated as separate factors playing roles in AMD pathology in previous studies (Wei et al. 2012; Zhou et al. 2010; Zipfel et al. 2010); in addition, it is the first time that GSK3 is suggested to play pathological roles in AMD (Figure 6).

Because GSK3 is constitutively activated (Biondi and Nebreda 2003), maintenance of PI3K/Akt activity is required for GSK3 inhibition and human health. GSK3 is connected to outside environment of the cell via PI3K/Akt as PI3K is activated by many various types of cell receptor signaling (PI3 Kinase Akt Signaling, Cell Signaling Technology), which are modulated by alteration of environmental and/or genetic factors (Figure 6). The data indicate that overstimulation of receptors results in insensitivity of the pathway (PI3K/Akt/GSK3), and increase of GSK3 activity, not tallying with a doctrine: a stimulus activates PI3K, PI3K activates Akt and Akt represses GSK3 activity. A clinical example shows that people treated with hormones including GH belonging to GH family that activates PI3K/Akt (Liu et al. 2011) also increase adverse effects, such as diabetes and glucose intolerance (Blackman et al. 2002), which can be expounded by possible high GSK3 activity in those people after a certain period of the hormone administration. Decreasing usage of ligands, such as thyroxine (Denckla 1974) that activates Akt (Kuzman et al. 2005), also leads to less activated PI3K/Akt and higher GSK3 activity in cells. Therefore, through any of the following ways: overstimulation of PI3K/Akt, reduction of using native PI3K/Akt stimuli or inhibition of PI3K/Akt, release of GSK3 activity eventually and always happens (Figure 6). A stimulus exists persistently, such as high IL17RC expression in eye and peripheral blood cells in AMD patients (Wei et al. 2012), or is repeatedly administrated, such as GH (Blackman et al. 2002), leading to overstimulation of PI3K/Akt. Then, the cell response system to any stimuli associated with the pathway will be broken, and the cells will become insensitive. The data show that levels of pGSK3α undulate in VEGF-treated ARPE (Figure 5A), whereas a more severe damage of PI3K/Akt/GSK3 sensitivity by IL17RC transfection in ARPE, the same cell line (Figure 4A) suggests the latter overstimulation is stronger. Of the four overstimulation results, the data indicate the sequence of overstimulation-caused insensitivity: GSK3 insensitivity happens first, then Akt insensitivity, and finally PI3K insensitivity (Figures 4A, B and 5A, B and 6). Even if cells treated with VEGF in shorter terms (Figures 5A, B and 6), GSK3 insensitivity still first occurs. There are three overstimulation stages showing the insensitivity of the kinases, presented by their activities. Stage1: high GSK3, Akt and PI3K activities coincide (Figures 5A and B); stage 2: high GSK3, low Akt and high PI3K activities concur (Figures 4B and 5A, B); stage 3: high GSK3, low Akt and PI3K activities coexist (Figure 4A). Those phenomena are summarized in Figure 6. On the other hand, overstimulation-caused damage by the same method shows different degrees in different cell lines (Figures 4A and B), probably due to different expression levels of the related-proteins in the cell lines. Most overstimulated cells live as only about 15-25% decrease in growth/survival is detected, whereas the insensitive GSK3 levels are about 40-70% in the cells with IL17RC overexpression or VEGF treatment (Figures 4 and 5). However, the overstimulation does not affect ERK1 signaling, Wnt-signaling-induced expression of c-Myc and cyclin D1, and/or β-catenin and VEGF (Figures 1, 4 and 6) so that the sensitivity of PI3K/Akt/GSK3 is vulnerable and different types of signaling are unsynchronized. ERK activation is dependent on PI3K/Akt by short stimulation (Venkatachalam et al. 2008), whereas it is intriguing that ERK activation is associated with high GSK3 activity during overstimulation stages when PI3K/Akt activities are abolished or reduced (Figure 4A). Treated with androgen and LY compound, a PI3K inhibitor for three-day, cells in which GSK3 activity is suggested to increase because of suppressed Akt activation dramatically augment ERK activation (Liu et al. 2011). Producing cells that scarcely respond to stimuli, overstimulation is suggested to be the fountainhead of the decreased use of hormones by the body cells. In this regard, any PI3K/Akt activators, which are unnecessary from the pituitary gland (Denckla 1974) and can be inorganic molecules, organic molecules or large molecules, can become death hormones as long as they overstimulate PI3K/Akt in the body cells. Moreover, according to the data, the alternative death hormone explanation suggests that the inhibition of the body cells using thyroxine and GH is due to past damage of PI3K/Akt/GSK3 sensitivity but not due to present suppression of the cell ability using the hormones (Denckla 1974).

Although many human cells sooner or later undergo normal apoptosis, and new cells will replace the apoptotic cells by the body regeneration functions (Carlson 2007), overstimulation-consequent high GSK3 activity-induced apoptosis, which is abnormal death, decreases cell growth/survival (Figures 4 and 5); moreover, high GSK3 activity damages cell functions in ways such as inactivating survival-promoting factors (Grimes and Jope 2001) and IRS1 (Figures 4 and 5); furthermore, overstimulation causes PI3K/Akt to be insensitive to environment stimuli (Figures 4 and 5), suggesting that overstimulation creates problems in many life processes including renewal of the body cells. In addition, the regeneration functions decrease with age; for instance, diminished epidermal cell proliferation is age-related (Grove and Kligman 1983). Hence, one’s health situation will become worse and worse since overstimulation events-caused insensitivity cells increase, whereas the body’s self-renewal ability to replace the insensitivity cells decreases, with age. On the other hand, the data suggest that high levels of some activators remain in the body cells. IL17RC increases IL17 expression and *vice versa* (Figures 1 and 6), suggesting that high expression of IL17RC is transferable between cells, especially from old cells to new cells with normal IL17RC expression via high concentrations of IL17 in the tissue environments (Wei et al. 2012). IL17 is also a PI3K activator (Chen et al. 2011). That is perhaps one of reasons why AMD is irreversible because high levels of IL17 and IL17RC which generate insensitivity kinases still exist despite cell replacement.

Accumulation of the insensitivity kinases in different tissues/organs is also unsynchronized since overstimulation is unevenly distributed and abilities for renewal of the body cells in different tissues/organs differ, illustrating why aging of the body tissues/organs differs. Diseases may start from local aging of the body tissues/organs. Inflammation is a common past or current symptom and associates with many seemingly unrelated chronic diseases (Ferrero-Miliani et al. 2007). Inflammation is caused by proinflammatory cytokine, which is the name used to describe a diverse group of soluble proteins, peptides, and glycoproteins that act as hormonal regulators or signaling molecules at nano- to-picomolar concentrations and help in cell signaling (Gilman et al. 2001). Inflammation is associated with high GSK3 activity (Jope et al. 2007). This study demonstrates that overstimulation increases GSK3 activity and suggests that high levels of proinflammatory cytokine causing inflammation linked with high GSK3 activity, one of the overstimulation consequences. Therefore, high levels of proinflammatory cytokine including IL17 (Venkatachalam et al. 2008) are likely to cause overstimulation in the body cells, and inflammation is an additional consequence of overstimulation. IL17 stimulating inflammatory response requires IL17RC (Venkatachalam et al. 2008). Overexpression of IL17RC also associates with inflammation, such as ocular sarcoidosis (Wu et al. 2014). Abnormally high GSK3 activity associates with type II Diabetes and Alzheimer’s disease (Wagman and Nuss 2001), whereas type II Diabetes is associated with AMD (Topouzis et al. 2009), and blinding AMD may be associated with cognitive impairment (Baker et al. 2009). All three diseases are associated with inflammation (Gilman et al. 2001; Wang et al. 2011). In cultured cells transfected with IL17RC or treated with VEGF , high levels of pIRS1, associating with type II Diabetes and causing insulin resistance (Liberman and Eldar-Finkelman 2005) (Figure 6) in that the body cells reduce their use of insulin (Kumar et al. 2005), and pTAU, associating with Alzheimer’s disease (Yang et al. 1999), have been detected (Figures 4 and 5). Increased PI3K/Akt/GSK3 insensitivity (Figure 6) can also decrease using insulin, which is another explanation of insulin resistance in addition to high levels of pIRS1, since insulin is also a PI3K activator (Brown et al. 1999). Logically, high levels of pIRS1 should appear after high levels of GSK3 insensitivity and activity in an over-stimulated cell. Additionally, GSK3β mediating high glucose-induced ubiquitination and proteasome degradation of IRS1 enhances insulin resistance (Leng et al. 2010). Moreover, AMD doubles heart attack and stroke risk (Tan et al. 2008). Immune cells with high GSK3 activity may also increase risk of cardiovascular diseases (CVD) and cancer since the cells are suggested to be apoptotic (Jope & Johnson 2004; Watcharasit et al. 2002; Grimes and Jope 2001) or insensitive to PI3K/Akt stimuli (Figures 4 and6) or with impaired cell functions (24, Figures 4 and 6); hence, these cells may have low capabilities to eliminate LDL particles and cell debris from blood vessels and to destroy abnormal cells. Whether high GSK3 activity and/or inactivation of PI3K/Akt in peripheral blood cells increase CVD or cancer risk or poor outcome of cancer remains to be investigated.

The study first elucidates how GSK3 activity is released and how cells become insensitive as well as die early, explaining why the body cells reduce use of the hormones with age (Denckla 1974; Blackman et al. 2002). Overstimulation-caused PI3K/Akt/GSK3 insensitivity and high GSK3 activity are the fundamentals to propose the alternative death hormones hypothesis. Now, the hypothesis not only is a testable model but also provides a rationale to interpret many clinical phenomena. Given that PI3K/Akt/GSK3 expression is in ubiquity and that the pathway is associated with almost any signaling, overstimulation-induced insensitivity in this study can occur in any cell of the body, which will in turn cause health problems. Biological mechanisms of many factors including diseases, genetic changes, habits, life styles, medical and psychological effects, health products, physical exercise, performance-enhancing drugs, obesity and/or hyperlipidemia, and hyperglycemia etc., are likely linked to the pathway.

## Electronic supplementary material

Additional file 1: Figure S1: Statistic results of some blots in Figures 1 and 4. A-C, Measurement of bands intensity in Figures 1B, D or Figure 4A. Intensity values from EV-cells are set as one. Data are averaged from three or more blots including shown ones in Figures 1B **(Figure S1A)**, D **(Figure S1B)** or Figure 4A **(Figure S1C)**, represented as mean ± SD. A, * versus EV of the same groups: p < 0.05. B, * versus control in the same groups: p <0.05; In c-Myc and cyclin D1 columns: there is no statistic difference between column 3 and column 1 or 2; # or ## versus any other treatment in the cognate groups: p < 0.05 or 0.01. C. *, ** or *** versus any other treatment in the same groups: p < 0.05, 0.01 or 0.001. (PDF 963 KB)

Additional file 2: Figure S2: Statistic results of some blots in Figures 4B and 5A. A-C, Measurement of bands intensity in Figures 4B and 5A. Intensity values from EV-cells (Figure 4B) or cells at 0 h (Figure 5A) are set as one. Data are averaged from three or more blots including shown ones in Figures 4B **(Figure S2A)** or 5A **(Figure S2B and C)**, represented as mean ± SD. A, * or ** versus any other treatment in the same groups: p < 0.05 or 0.01. B and C, * or ** versus control in the same groups: p < 0.05 or 0.01. (PDF 1 MB)

Additional file 3: Figure S3: Statistic results of some blots in Figures 5B. A and B, Measurement of bands intensity in Figure 5B. Intensity values from cells at 0 h (Figure 5B) are set as one. Data are averaged from three or more blots (Figure 5B) including shown blots in Figure 5B, represented as mean ± SD. * or ** versus control in the same groups: p < 0.05 or 0.01. (PDF 558 KB)
