# Supplementary figures and images for: Overstimulation can create health problems due to increases in PI3K/Akt/GSK3 insensitivity and GSK3 activity
Source: Springerplus. 2014 Jul 14;3:356. doi: 10.1186/2193-1801-3-356 (PMC4117863; doi:10.1186/2193-1801-3-356)

Figure S1

**A**

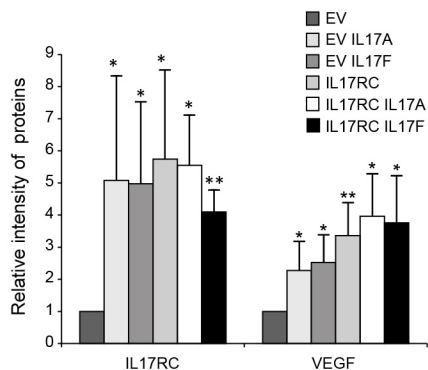

**B**

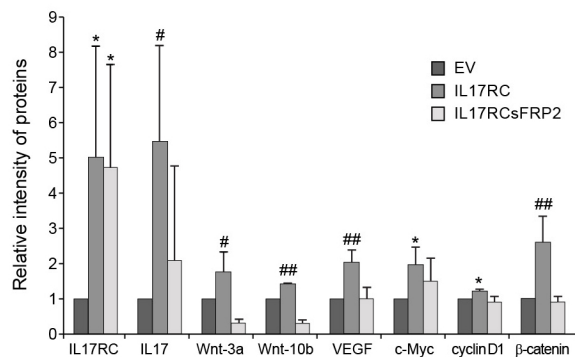

**C**

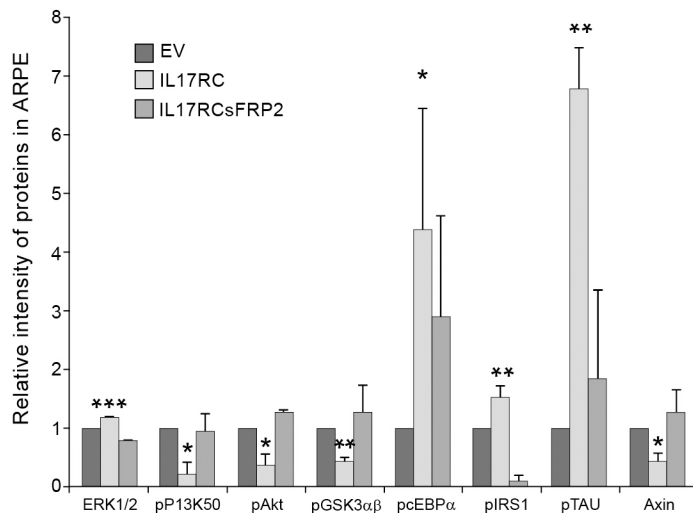

Supplement: Supplementary file 1 — Additional file 1: Figure S1: Statistic results of some blots in Figures 1 and 4. A-C, Measurement of bands intensity in Figures 1B, D or Figure 4A. Intensity values from EV-cells are set as one. Data are averaged from three or more blots including shown ones in Figures 1B (Figure S1A), D (Figure S1B) or Figure 4A (Figure S1C), represented as mean ± SD. A, * versus EV of the same groups: p < 0.05. B, * versus control in the same groups: p <0.05; In c-Myc and cyclin D1 columns: there is no statistic difference between column 3 and column 1 or 2; # or ## versus any other treatment in the cognate groups: p < 0.05 or 0.01. C. *, ** or *** versus any other treatment in the same groups: p < 0.05, 0.01 or 0.001. (PDF 963 KB) [file 40064_2014_1083_MOESM1_ESM.pdf]

Figure S2

**A**

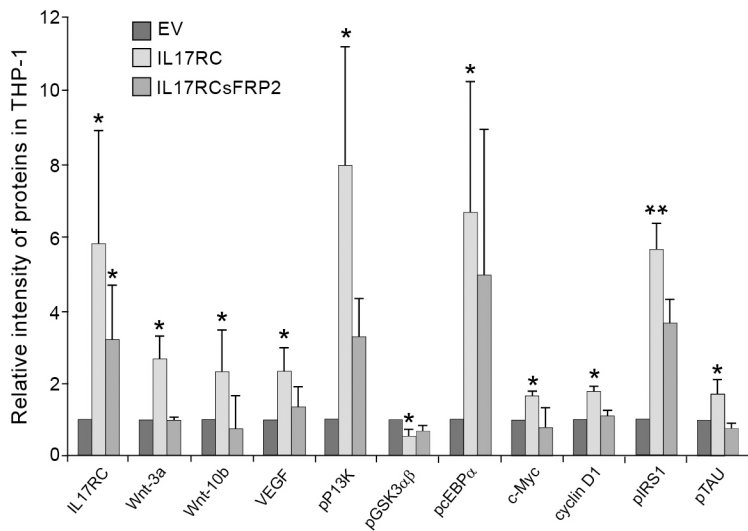

**B**

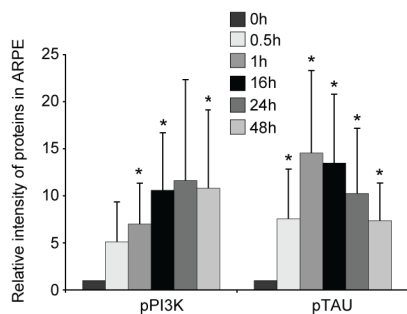

**C**

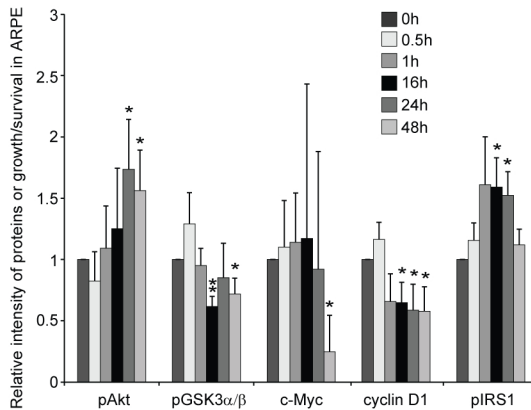

Supplement: Supplementary file 2 — Additional file 2: Figure S2: Statistic results of some blots in Figures 4B and 5A. A-C, Measurement of bands intensity in Figures 4B and 5A. Intensity values from EV-cells (Figure 4B) or cells at 0 h (Figure 5A) are set as one. Data are averaged from three or more blots including shown ones in Figures 4B (Figure S2A) or 5A (Figure S2B and C), represented as mean ± SD. A, * or ** versus any other treatment in the same groups: p < 0.05 or 0.01. B and C, * or ** versus control in the same groups: p < 0.05 or 0.01. (PDF 1 MB) [file 40064_2014_1083_MOESM2_ESM.pdf]

Figure S3

**A**

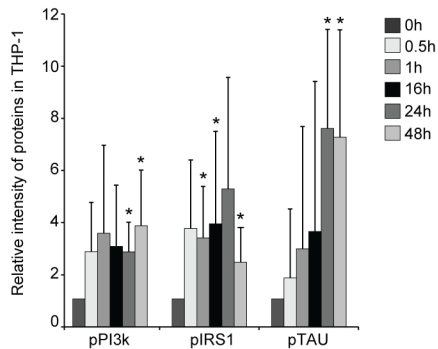

**B**

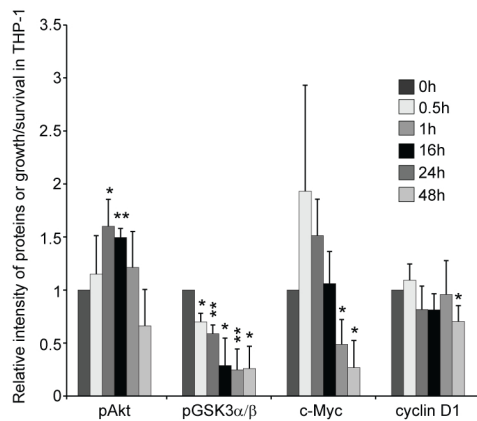

Supplement: Supplementary file 3 — Additional file 3: Figure S3: Statistic results of some blots in Figures 5B. A and B, Measurement of bands intensity in Figure 5B. Intensity values from cells at 0 h (Figure 5B) are set as one. Data are averaged from three or more blots (Figure 5B) including shown blots in Figure 5B, represented as mean ± SD. * or ** versus control in the same groups: p < 0.05 or 0.01. (PDF 558 KB) [file 40064_2014_1083_MOESM3_ESM.pdf]
